# Thematic Selection: Stress and Stress-related Disorders Developmental and Neuroendocrine Aspects of Stress and Stress-related Disorders (Part 1)

**DOI:** 10.2174/1570159X2203231024142551

**Published:** 2023-10-24

**Authors:** Agorastos Agorastos

**Affiliations:** 1 Assistant Professor of Psychiatry, Aristotle University of Thessaloniki, Greece

Stress is defined as the state of threatened homeodynamic balance of the organism [1]. To preserve such an optimal homeodynamic state, organisms have developed a highly sophisticated neuroendocrine system, the stress system, which serves self-regulation and adaptability by energy redirection and coordination of subordinate homeostatic systems (*i.e*., immune, metabolic, cardiovascular) according to the current needs [2, 3].

The human stress system includes central and peripheral components. The central components are located in the hypothalamus and the brainstem and interact with several major brain nuclei and neuromodulatory systems, such as the mesocortical and mesolimbic dopaminergic system (reward and motivation), the amygdalae (fear, anger and arousal), the endocannabinoid system (cognitive and physiological regulation) and the central circadian system (temporal organization) [4]. The peripheral components include the hypothalamic-pituitary-adrenal (HPA) axis and the limbs of the autonomic nervous system (ANS), which comprises the sympathetic nervous and sympatho-adrenomedullary (SNS, SAM) system and the parasympathetic nervous system. The principal peripheral effector molecules of the stress system are the HPA axis end-hormones, the glucocorticoids (GCs; *i.e*., cortisol in humans), and the SAM-regulated catecholamines norepinephrine and epinephrine/adrenaline. GCs exert their pleiotropic effects mainly through genomic, nongenomic and mitochondrial actions *via* glucocorticoid and mineralocorticoid receptors [5]. Proper coordination of these homeostatic systems is essential to individual development, adaptation, survival, and well-being, whereby neuroendocrine responses to stress depend on developmental timing, duration, time of day, nature of stressors and subjective appraisal [6].

Inadequate, excessive or chronic (*i.e*., prolonged, persistent or repeated) stress responses, especially in stress-sensitive developmental stages of higher brain plasticity and increased epigenetic sensitivity (*e.g*., early childhood), may surpass the organism’s natural adaptive capacity and negatively alter neuroendocrine responses to stress [7]. Early life stress, in particular, can disrupt developmental programming of the related neural circuitry and lead to over-/undersensitization-related alterations in neuroendocrine, immune, circadian, emotional and autonomic (re-)activity, with impaired GC signaling and related structural, functional and epigenetic modifications in both the brain and peripheral tissues [4, 8-12]. Such changes may trigger health-related risk cascades and represent a common developmental risk factor and cumulative health risk mediator in the continuum of chronic disease development, leading to a vulnerable biological phenotype with increased physical and mental morbidity in later life [4, 13-15].

Acute and chronic dysregulation of the stress system at different levels has been implicated as a major downstream pathway and linked to a broad range of complex behavioural (*e.g*., anxiety, depression, eating disorders, post-traumatic stress disorder, sleep disorders) and somatic disorders (*e.g*., chronic pain and fatigue syndromes, obesity, metabolic syndrome, chronic inflammation, diabetes type II, hypertension, atherosclerosis, cardiovascular diseases, body composition disorders cancer), curtailing life expectancy [4, 16-18]. In fact, chronic stress is suggested as a common risk factor for 75-90% of chronic, non-communicable diseases [19]. However, although much research is taking place in the field of acute and chronic stress, less is known about the transitional phase, in which acute stress becomes chronic and gets “under the skin” to influence disease development [20].

This special thematic selection entitled “Stress and stress-related disorders” consists of three individual issues with selected review articles of expert research groups. The current issue (Part 1) handles developmental and neuroendocrine aspects of stress and stress-related disorders.

A total of four review articles focus on developmental aspects of stress. The first two articles by Nicolaides *et al.* [21] and Filetti *et al.* [22] provide a multifaceted overview of the developmental neuroendocrinology in children and adolescents with a specific focus on the regulation of stress reactivity and child development and behavior. The next two articles by Xenaki *et al.* [23] and Cullen *et al.* [24] offer a developmental take on the role of stress and environment and the importance of stress-related research in the neurobiology of psychosis by two expert research groups in the field.

Another four review articles focus on neuroendocrine aspects of stress and stress-related disorders. Hodes *et al.* [25] provide an expert review on the very important and often neglected topic of gender-related differences in stress response mechanisms. The next two articles by Kulakova *et al.* [26] and Colizzi *et al.* [27] investigate the interaction between the HPA axis and social cognition as well as the endocannabinoid system in the examples of borderline personality disorder and psychosis, respectively. Finally, Bendau *et al.* [28] review the neuroendocrine mechanisms and the treatment effects of exercise in different stress-related disorders [28].

Understanding the pathways susceptible to disruption following stress exposure could provide new insights into the pathophysiologic trajectories that link toxic stress during developmental stages to adult maladjustment and psychopathology and help us develop new translational diagnostic approaches and revised, neurobiology-driven treatment modalities.
